# Recombinant ArtinM activates mast cells

**DOI:** 10.1186/s12865-016-0161-0

**Published:** 2016-07-04

**Authors:** Valéria Cintra Barbosa-Lorenzi, Nerry Tatiana Cecilio, Patricia Andressa de Almeida Buranello, Maria Cristina Pranchevicius, Maria Helena S. Goldman, Gabriela Pereira-da-Silva, Maria Cristina Roque-Barreira, Maria Célia Jamur, Constance Oliver

**Affiliations:** Department of Cell and Molecular Biology and Pathogenic Bioagents, Ribeirão Preto Medical School, University of São Paulo, Ribeirão Preto, SP Brazil; Department of Biology, Faculdade de Filosofia Ciências e Letras de Ribeirão Preto, Universidade de São Paulo, Ribeirão Preto, SP Brazil; Department of Maternal-Infant Nursing and Public Health, Escola de Enfermagem de Ribeirão Preto, Universidade de São Paulo, Ribeirão Preto, SP Brazil; Present address: Department of Biochemistry, Weill Cornell Medical College of Cornell University, New York, NY USA; Present address: Department of Biological Sciences, Universidade Federal do Triangulo Mineiro, Uberaba, MG Brazil; Present address: Department of Genetics and Evolution, Universidade Federal de São Carlos, São Carlos, SP Brazil

**Keywords:** Mast cells, rArtinM, ArtinM, Degranulation, Lectin

## Abstract

**Background:**

Mast cells are hematopoietically derived cells that play a role in inflammatory processes such as allergy, as well as in the immune response against pathogens by the selective and rapid release of preformed and lipid mediators, and the delayed release of cytokines. The native homotetrameric lectin ArtinM, a D-mannose binding lectin purified from *Artocarpus heterophyllus* seeds, is one of several lectins that are able to activate mast cells. Besides activating mast cells, ArtinM has been shown to affect several biological responses, including immunomodulation and acceleration of wound healing. Because of the potential pharmacological application of ArtinM, a recombinant ArtinM (rArtinM) was produced in *Escherichia coli.* The current study evaluated the ability of rArtinM to induce mast cell degranulation and activation.

**Results:**

The glycan binding specificity of rArtinM was similar to that of jArtinM. rArtinM, via its CRD, was able to degranulate, releasing β-hexosaminidase and TNF-α, and to promote morphological changes on the mast cell surface. Moreover, rArtinM induced the release of the newly-synthesized mediator, IL-4. rArtinM does not have a co-stimulatory effect on the FcεRI degranulation via. The IgE-dependent mast cell activation triggered by rArtinM seems to be dependent on NFkB activation.

**Conclusions:**

The lectin rArtinM has the ability to activate and degranulate mast cells via their CRDs. The present study indicates that rArtinM is a suitable substitute for the native form, jArtinM, and that rArtinM may serve as an important and reliable pharmacological agent.

## Background

Mast cells are hematopoietically derived cells that reside in the connective tissue [1–5] and play a major role in the immune response in both physiological and pathological processes such as allergy, inflammation, cardiac disease, cancer, autoimmune diseases, and wound healing [6–9]. The activation of mast cells in these processes results in degranulation, release of lipid mediators and cytokines [10]. The mast cell mediators then recruit other cell types including T lymphocytes, neutrophils, and dendritic cells to inflammatory sites [11–13].

Lectins are among the diverse molecules that have been shown to activate mast cells [14–16]. It has previously been shown that the native tetrameric ArtinM (jArtinM), a D-mannose-binding lectin from *Artocarpus heterophyllus* (jackfruit) seeds, induces the recruitment of rat mast cells from bone marrow to the peritoneal cavity [17], as well as inducing degranulation of rat peritoneal mast cells [11]. In the rat mast cell line RBL-2H3, jArtinM stimulates NFAT (nuclear factor of activated T-cells) and NFkB (nuclear factor kappa-light-chain-enhancer of activated B cells) in an IgE independent manner [18]. In addition to its action on mast cells, jArtinM also recruits neutrophils [19] by binding to glycans of CXCR2 that stimulate signal transduction via G protein [20], thus activating the cells and increasing their phagocytic activity against pathogens [21]. jArtinM also has immunomodulatory activity. Systemic administration of jArtinM confers protection against intracellular parasites such as *Leishmania major* and *Paracoccidioides brasiliensis*, by inducing IL-12 production through interaction with TLR2 N-glycans, resulting in a Th1-type immune response [22, 23].

A recombinant form of jArtinM, rArtinM, has been heterologously expressed in *Escherichia coli* [24, 25]. rArtinM is produced as soluble monomers with its CRDs preserved and active [25]. Furthermore, the binding affinity of rArtinM to the trimannoside Manα1-3 [Manα1-6] Man from HRP, a N-glycosylated protein, is similar to the native form [26]. Additionally, rArtinM showed both prophylactic and therapeutic effects during the course of *P. brasiliensis* infection in mice [27]. The present investigation was undertaken to evaluate if rArtinM, as a monomeric molecule, has the same ability as jArtinM to activate mast cells. In the current study, rArtinM was shown to have the same binding affinity to N-glycans as the native form, jArtinM, and was also able to activate and degranulate mast cells through its CRDs.

## Results

### Analysis of rArtinM

**Fig. 1:**
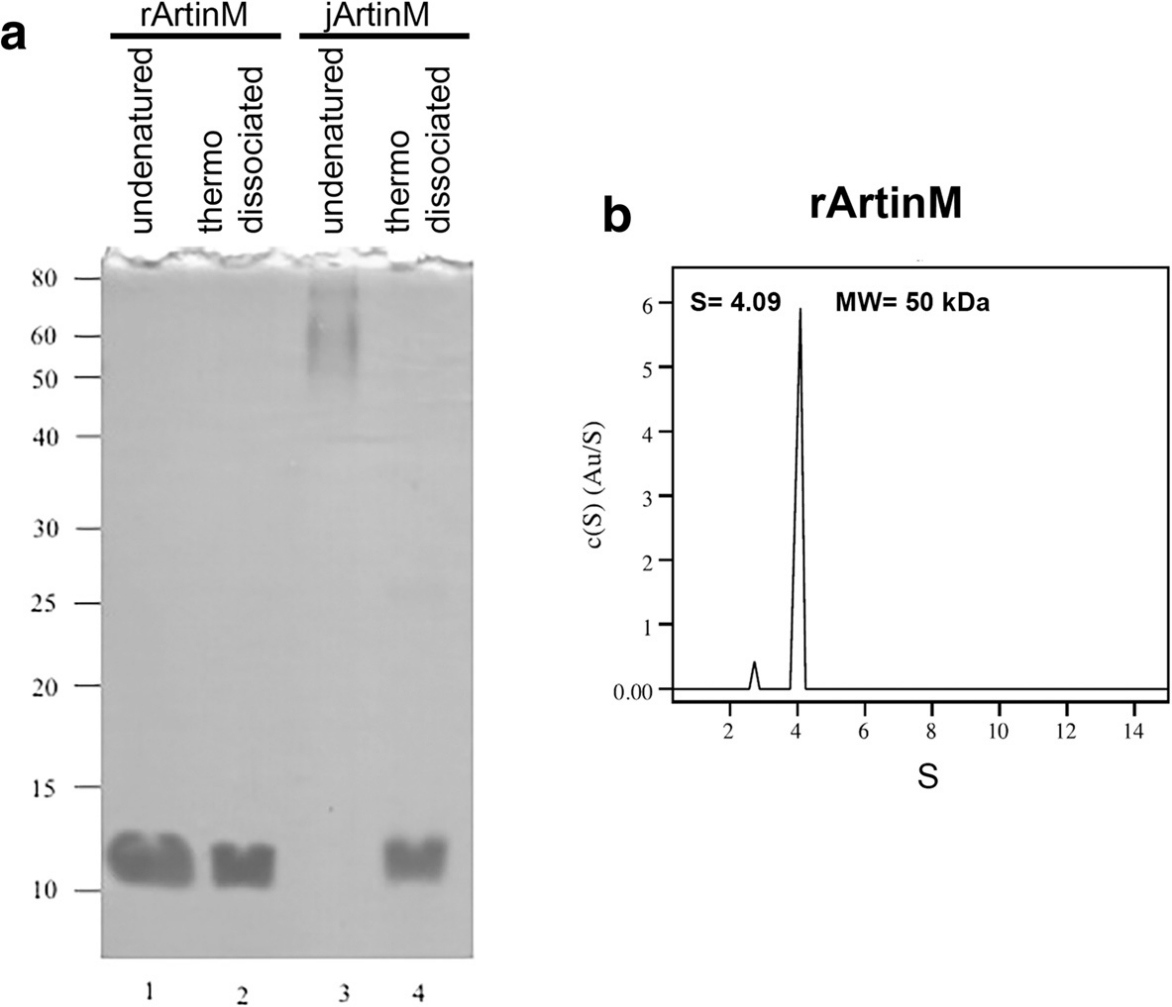
Analysis of rArtinM and jArtinM and analytical ultracentrifugation assay. **a** Lane 1: undenatured rArtinM. Lane 2: rArtinM after thermal dissociation. Lane 3: undenatured jArtinM. Lane 4: jArtinM after thermal dissociation. 3 μg of protein were loaded to each lane. 12.5 % SDS-PAGE stained with Coomassie blue G-250. **b** Size distribution obtained from the sedimentation velocity profiles of rArtinM at 20 °C. Fit and residuals after fitting to a c(S) were calculated in SEDFIT. Plot of the distribution of sedimentation coefficients (*C*(s)) versus (S), where S is plotted in Svedberg units. The shape of the major peak shows oligomeric structures of rArtinM. By non-linear fitting, the average molecular weight (Mw) for rArtinM was determined to be 50 kDa (displayed inside the plot)

The objective of the present study was to characterize the effect of monomeric rArtinM on mast cells. Therefore, it was essential to confirm that rArtinM was indeed monomeric. SDS-polyacrylamide gel electrophoresis (SDS-PAGE) was used to compare the homogeneity of native and recombinant ArtinM preparations. Under nondenaturing conditions or after thermal dissociation rArtinM exhibited a single protein band of approximately 13 kDa that corresponds to rArtinM monomers (Fig. 1a, lanes 1 and 2). jArtinM, the native tetrameric form, was used as a control. When undenatured jArtinM was loaded onto the gel, a protein band of approximately 60–80 kDa was observed. This band corresponds to jArtinM tetramers (Fig. 1a, lane 3). When jArtinM was submitted to thermal dissociation, a single protein band of approximately 13 kDa, corresponding to the dissociated tetramers (Fig. 1a, lane 4), was observed. These results indicate that *E. coli* expresses a monomeric form of ArtinM. It is also plausible that *E. coli* expresses oligomeric forms of ArtinM but these forms cannot be detected by electrophoresis, since their bonds could be dissociated by exposure to SDS.

jArtinM and rArtinM were also submitted to size exclusion chromatography on a Superdex 75 column, which was calibrated by using protein molecular weight standards. jArtinM presented two distinct peaks, the first with the apparent molecular mass of 42 kDa and the second peak with the apparent molecular mass of 22 kDa, together these two peaks had a molecular mass of 64 kDa (Table 1). This estimate is compatible with previous data from mass spectrometry analysis [28]. rArtinM had the lowest molecular mass, 13 kDa, thus reinforcing the hypothesis that rArtinM is expressed in a monomeric form (Table 1).

**Table 1:** Estimate of the molecular weight by size exclusion gel filtration chromatography

Sample	Kav	MW
jArtinM (peak 1)	0.1077	41.6584
JArtinM (peak 2)	0.2184	22.8336
rArtinM	0.3214	13.0524

jArtinM and rArtinM were submitted to size exclusion chromatography on a Superdex 75 column. Eluted peaks were analyzed by retention volume and molecular weight was calculated by its partition coefficient (Kav). 41.6 kDa (jArtinM peak 1), 22.8 kDa (jArtinM peak 2), and 13 kDa (rArtinM)

Because both of these analytical techniques could result in the dissociation of weakly associated monomers, an analytical ultracentrifugation assay (AUC) was performed to determine if rArtinM monomers are able to self-associate in solution. The AUC analysis, based on the sedimentation velocity and also on the sedimentation equilibrium, showed that in solution, rArtinM appeared to be trimeric, and exhibited a molecular weight of approximately 50 kDa (Fig. 1b). Minor peaks were absent from rArtinM. A previous AUC analysis of jArtinM demonstrated that it has an S value of 4.24 and is a 64 kDa tetramer. However, jArtinM is capable of forming higher order oligomeric species since there were minor peaks at S values of approximately 6, 7, 9, 10 [29]. This is in agreement with previous results using molecular modeling [30]. To understand these differences, crystal trials are being conducted on the two ArtinM forms using the crystallization conditions published for native artocarpin [30].

The molecular weights obtained from the sedimentation velocities were confirmed by sedimentation equilibrium, which makes no assumption regarding the hydrodynamic shape of the protein. This analysis shows that, in solution, rArtinM monomers are able to self-oligomerize, mostly into trimers.

### jArtinM and rArtinM share carbohydrate binding specificity

**Fig. 2:**
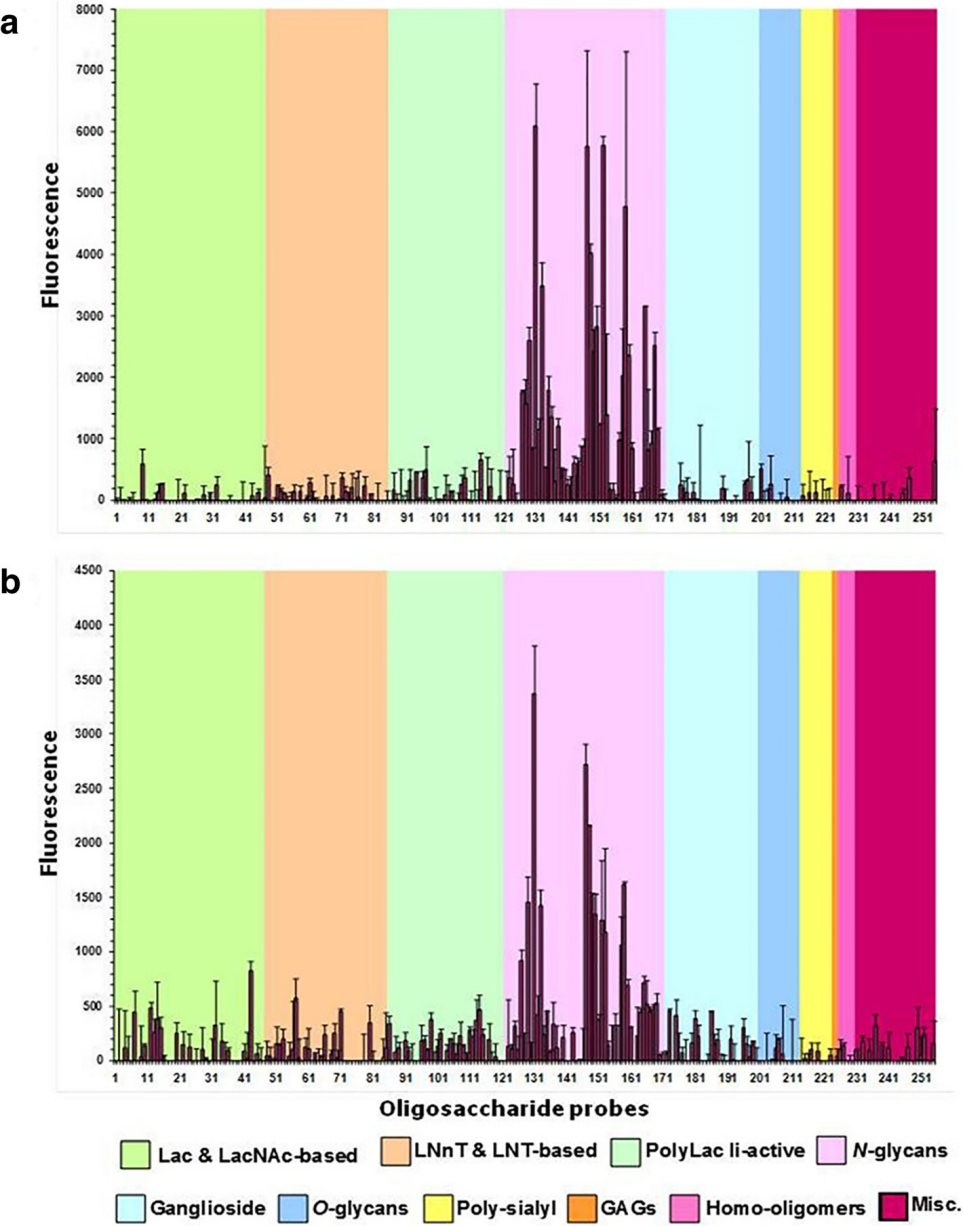
Carbohydrate microarray analyses of jArtinM and rArtinM. Binding of jArtinM (**a**) and rArtinM (**b**) to a glycan array of 255 lipid-linked oligosaccharide probes. Biotinylated ArtinM (50 μg/mL) was overlaid, and binding was detected after adding streptavidin conjugated to Alexa Fluor 647. Glycoarray data analysis was performed with dedicated software. Numerical scores of the binding signals are means of duplicate spots at 5 fmol/spot (with error bars). The various types of oligosaccharide linkage are indicated by the colored panels

It has been previously shown that rArtinM exhibits the same binding affinity for the trimannoside Manα1-3[Manα1-6] Man as does jArtinM. [25, 26] To assess the carbohydrate-binding properties of jArtinM and rArtinM at a higher resolution, glycan microarray analyses was carried out using a panel of 255 lipid-linked oligosaccharide probes representing diverse mammalian glycan sequences and their analogs. Fifty N-glycan related probes were included in the array. jArtinM (Fig. 2a) and rArtinM (Fig. 2b) bound exclusively to the N-glycan sequences, in agreement with published data on the specificity of the native form [31]. There was no significant difference in the specificities of the recombinant and native lectins, suggesting that the features that allow for the modified oligomerization of rArtinM do not affect the three-dimensional structure of its CRD, which is responsible for the glycan binding specificity.

### rArtinM induces mast cell degranulation and activation through its carbohydrate recognition domains (CRDs)

**Fig. 3:**
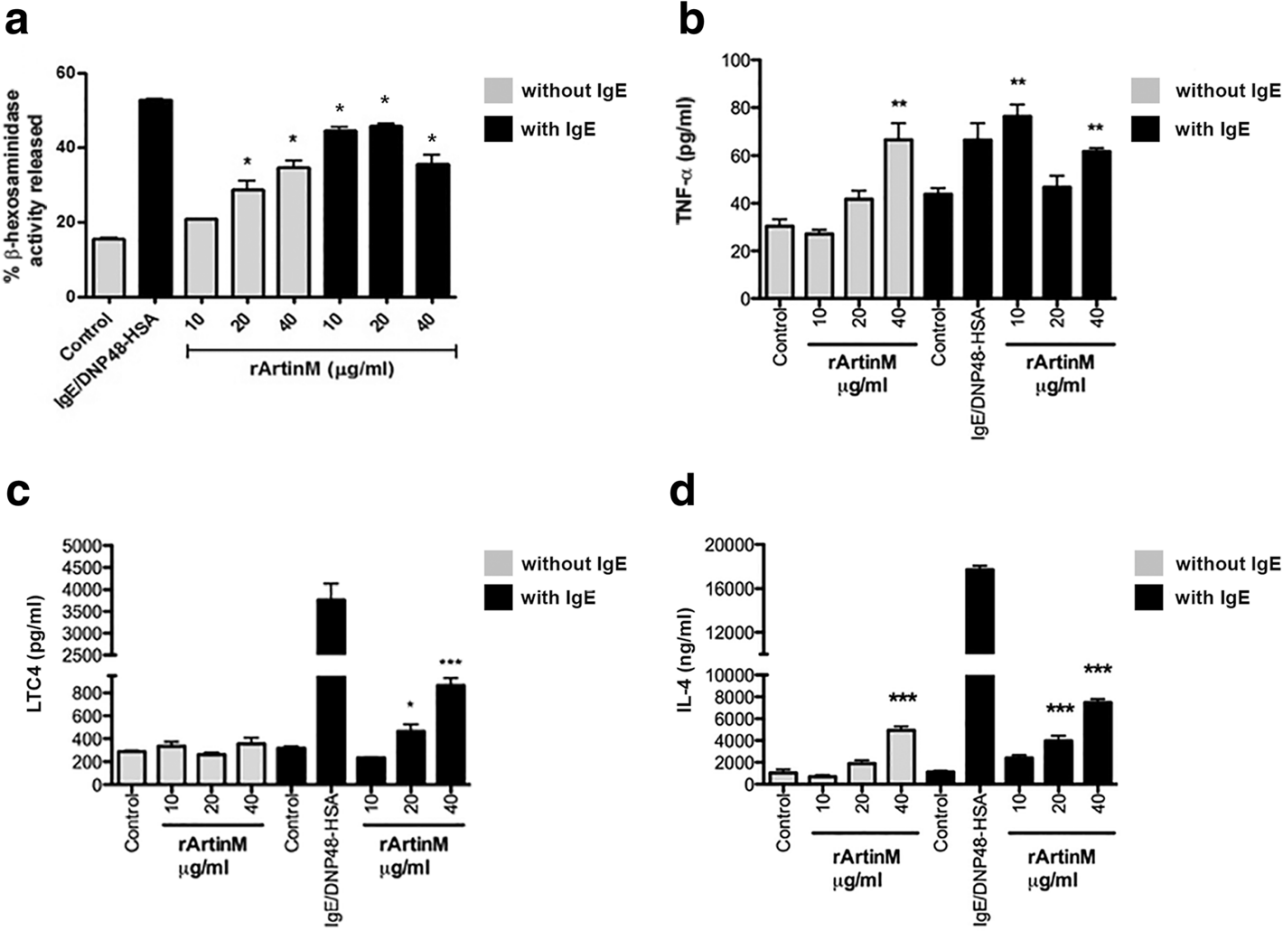
High doses of rArtinM are required for mast cell mediator release. **a** In the absence of IgE only 20 and 40 μg/ml of rArtinM induced β-hexosaminidase release, and in the presence of IgE all doses induced β-hexosaminidase release. **b** In the absence of IgE only 40 μg/ml of rArtinM stimulated release of TNF-α, whereas in IgE sensitized cells 10 and 40 μg/ml of rArtinM induced TNF-α release. **c** The release of LTC4 was seen only in IgE sensitized cells. **d** rArtinM stimulation of IL-4 release did not require IgE. Data are expressed as mean ± SEM and are representative of three separate experiments. *p < 0.05 or ** *p* < 0.01 or *** *p* < 0.001 between samples and the controls

The next step was to investigate if the recombinant form of ArtinM would be able to induce mast cell degranulation. A dose–response curve for the release of β-hexosaminidase and TNF-α was performed (Fig. 3). In the absence of IgE, only the higher concentrations of rArtinM were able to release β-hexosaminidase (20 and 40 μg/ml) and TNF-α (40 μg/ml) (Fig. 3a and b). On the other hand, in the presence of IgE, all concentrations of rArtinM induced β-hexosaminidase release (Fig. 3a), and for TNF-α there was a greater release at 10 and 40 μg/ml of rArtinM (Fig. 3b). The ability of rArtinM to induce the production of the newly formed lipid mediator, LTC4, was also examined. The production of LTC4 was observed only in sensitized cells and occurred in a dose-dependent manner (Fig. 3c). The synthesis of the newly-synthesized mediator, IL-4, was also investigated. Only the higher concentrations of rArtinM (20 and 40 μg/ml) induced the synthesis and release of IL-4 either in the presence or absence of IgE (Fig. 3d).

**Fig. 4:**
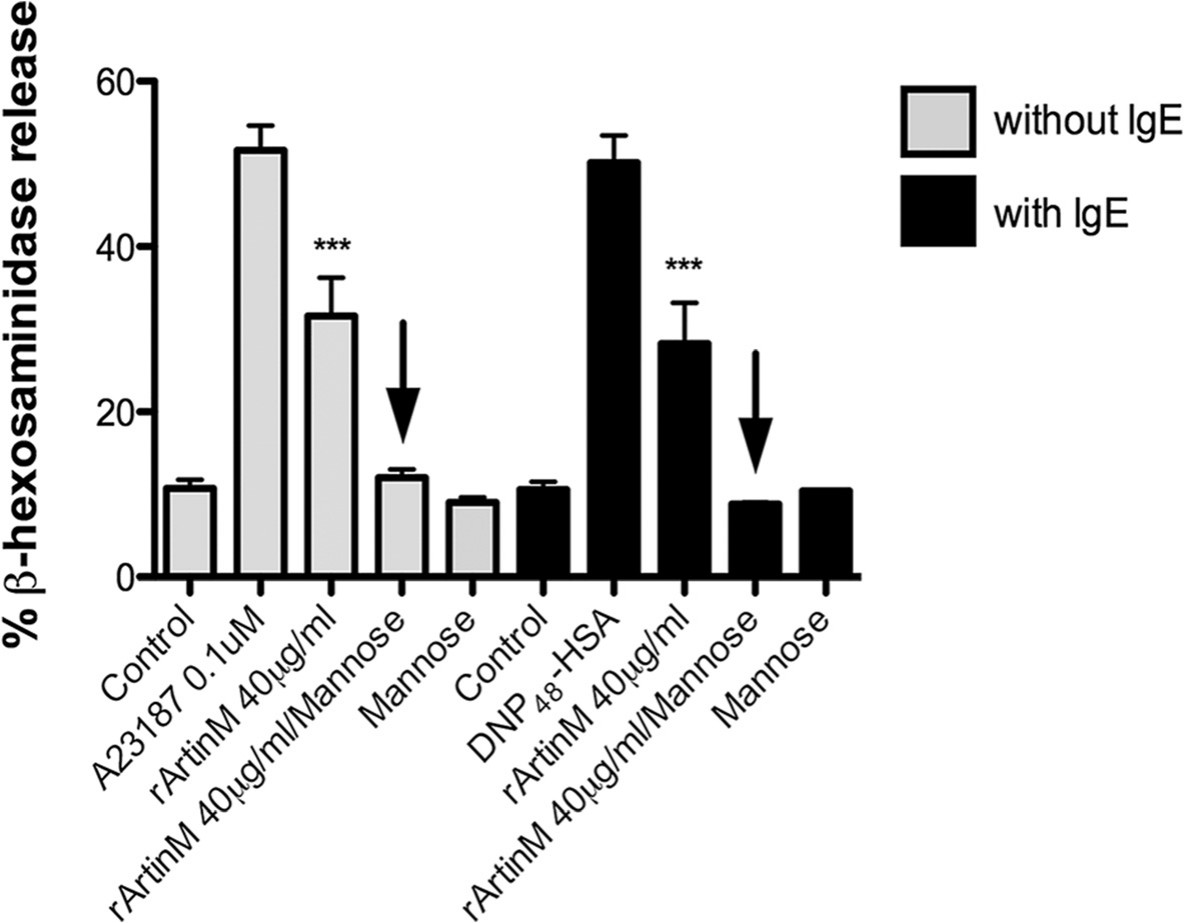
rArtinM induces degranulation via its CRDs. When rArtinM (40 μg/ml) was preincubated with D-mannose for 1 h, β-hexosaminidase release was abolished (black arrows). Data are expressed as mean ± SEM and are representative of three independent experiments. *** *p* < 0.001 between samples and the controls

**Fig. 5:**
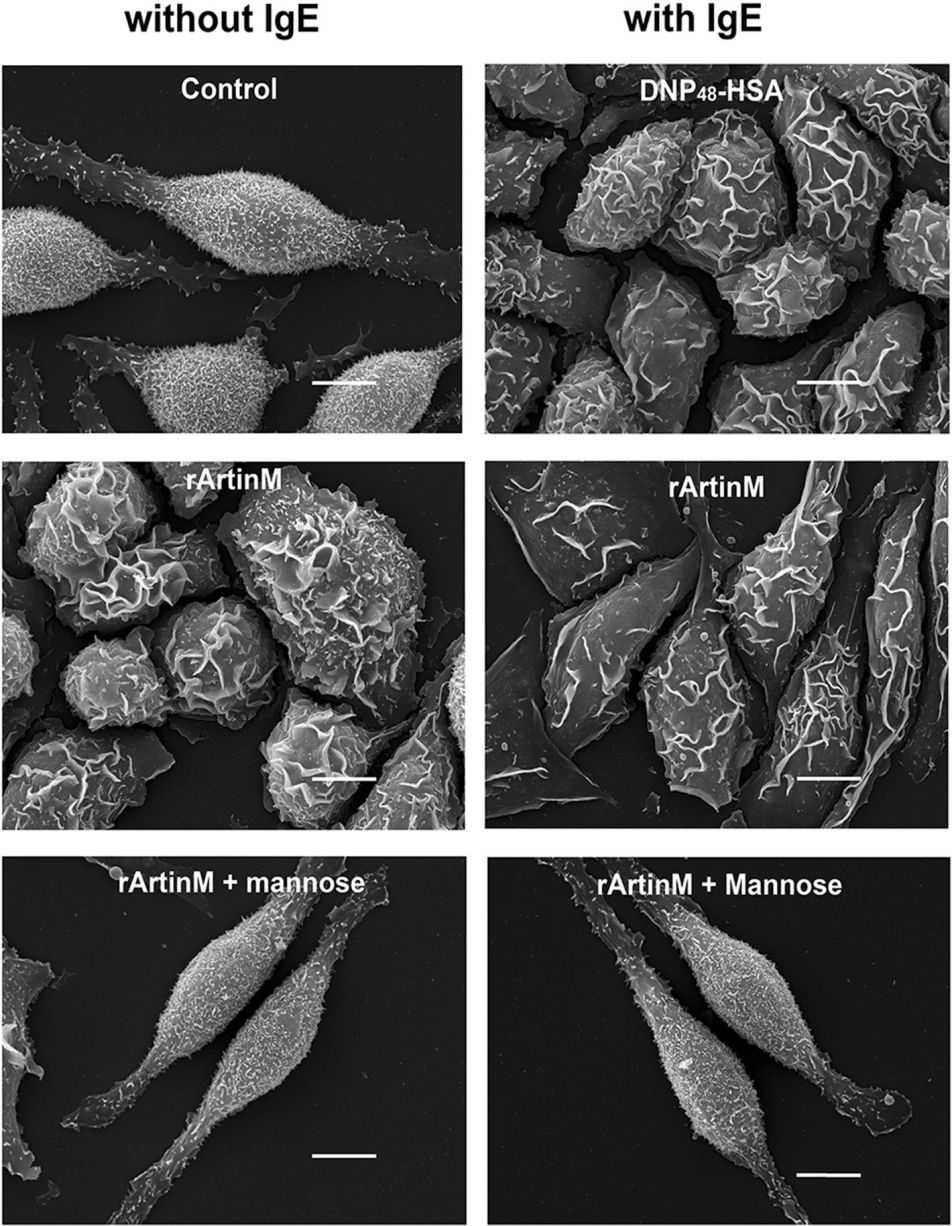
Activation induced by rArtinM occurs through a CRD-dependent manner. By scanning electron microscopy (SEM) deep ruffles are observed on the surface of IgE sensitized mast cells after activation with DNP_48_-HSA and on the surface of rArtinM treated mast cells both in the absence or presence of IgE. When rArtinM was preincubated with D-mannose, the cells are in spindle-shape and covered by fine microvilli on surface, similar to unstimulated cells (Control). Bars = 10 μm

It was then of interest to determine if the degranulation and activation induced by rArtinM was dependent on its CRDs. Therefore, rArtinM (40 μg/ml) was pre-incubated with its specific sugar, D-mannose, and β-hexosaminidase release assay was performed. Pre-incubation of rArtinM with D-mannose abolished the release of β- hexosaminidase, in the absence or presence of IgE (Fig. 4), indicating that mast cell degranulation mediated by rArtinM is dependent on its CRDs. The effect of rArtinM on mast cell activation was also assessed by scanning electron microscopy (SEM). By SEM unstimulated RBL-2H3 cells are spindle shaped and their surface is covered with fine microvilli (Fig. 5). After stimulation via FcεRI the cells have deep ruffles on their surface and are spread over the substrate (Fig. 5). Incubation with rArtinM (40 μg/ml) produced similar changes, which are consistent with mast cell activation (Fig. 5). When rArtinM (40 μg/ml) was pre-incubated with D-mannose, the cells had the same morphological characteristics as the unstimulated cells (Fig. 5). Taken together, these results show that mast cell activation and degranulation induced by rArtinM is mediated by its CRDs.

### rArtinM does not have a co-stimulatory role in mast cell degranulation via FcεRI

**Fig. 6:**
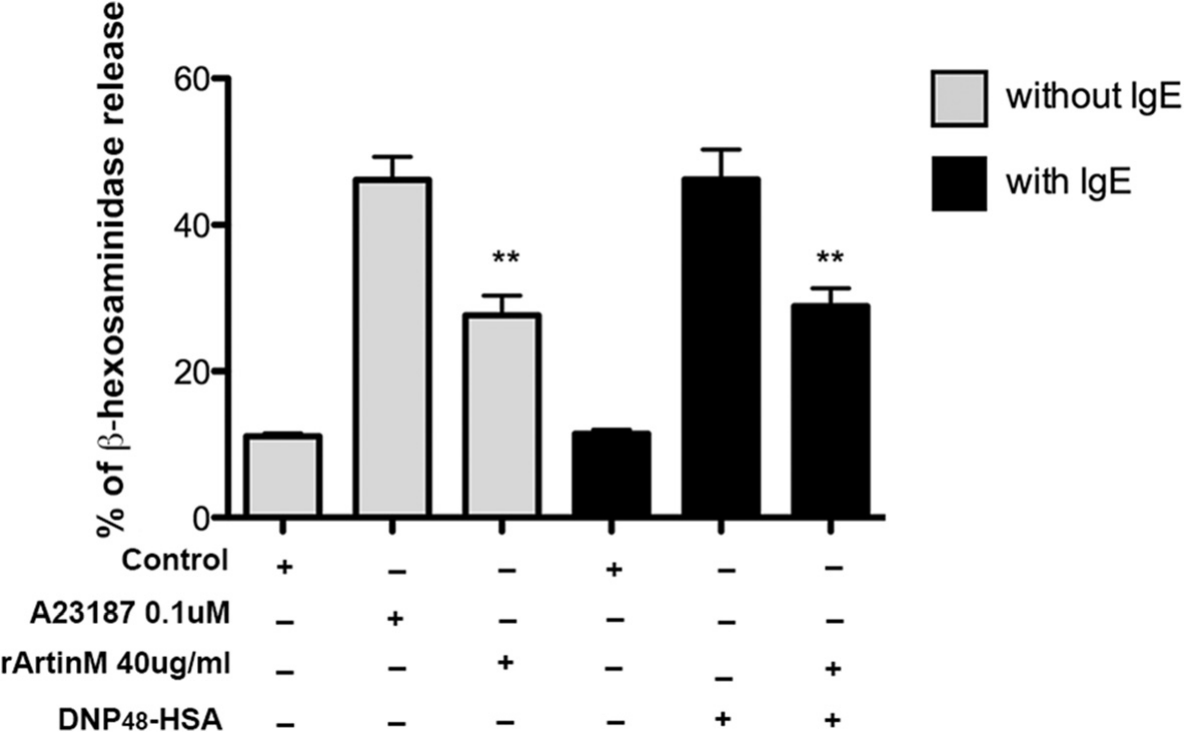
rArtinM does not have co-stimulatory effect on mast cell degranulation via FcεRI. Cells sensitized with IgE and stimulated with the combination of DNP_48_-HSA + rArtinM (40 μg/ml) had a decrease in β-hexosaminidase release. Data are expressed as mean ± SEM and are representative of three separate experiments. ** *p* < 0.01 between samples and the controls

Since the recombinant form of ArtinM is produced as monomers, it was of interest to investigate if rArtinM could act as a co-stimulatory molecule in the FcεRI signaling pathway. Co-stimulatory molecules act in parallel with a major signaling pathway, either by amplifying the intensity of the stimuli or by extending/prolonging its time-response [32]. For this, rArtinM was incubated for 30 min along with the IgE-TNP specific antigen, DNP_48_-HSA, and β-hexosaminidase release assay was performed. Interestingly, when rArtinM was jointly incubated with DNP_48_-HSA, the levels of β-hexosaminidase released were diminished by 37 % (Fig. 6) compared to cells stimulated with DNP_48_-HSA alone, suggesting that rArtinM disrupts the IgE-DNP_48_-HSA binding, probably blocking the interaction between DNP_48_-HSA and IgE.

### rArtinM activates the transcription factor NFkB

**Fig. 7:**
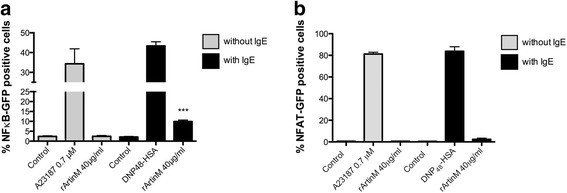
rArtinM activates NFkB, but not NFAT. rArtinM (40 μg/ml) induced NFkB activation only in the presence of IgE (**a**), but did not activate the transcription factor NFAT either in the absence or in the presence of IgE (**b**). Data are expressed as mean ± SEM and are representative of three separate experiments. *** *p* < 0.001 between samples and the controls

Since rArtinM was able to activate and degranulate mast cells, it was of interest to investigate if rArtinM is activating transcription factors such as NFkB and NFAT. For this, transfected RBL-2H3 cell lines with a GFP-gene reporter for NFkB and NFAT expression were used. rArtinM was able to activate NFkB in an IgE-dependent manner, but rArtinM did not activate NFAT (Fig. 7).

## Discussion

The present study shows that the recombinant monomeric form of ArtinM, rArtinM, expressed in *Escherichia coli*, has the ability to induce mast cell activation and degranulation. Activation by rArtinM resulted in the release of preformed, newly formed, and newly-synthesized mediators. Also, mast cell activation triggered by rArtinM results in morphological changes that are characteristic of activated mast cells. Furthermore, mast cell activation induced by rArtinM is dependent on its CRDs. Additionally, the IgE-dependent activation of mast cells triggered by rArtinM is dependent on NFkB activation.

The ubiquitous distribution of mast cells places them in a privileged position to act as sentinel cells, responding rapidly to external signals by releasing their stored preformed mediators and secreting newl*y*-synthesized lipid mediators [10]. One of the principal preformed mediators released during mast cell degranulation is TNF-α [6, 33]. TNF-α is an important chemoattractant for neutrophils and T cells during inflammatory processes [11, 34–36]. Besides TNF-α, leukotrienes also play a role in recruiting neutrophils and T cells to sites of inflammation [7, 37]. The release of TNF-α and LTC4 induced by rArtinM may help explain some of the biological activities attributed to this lectin such as accelerated tissue regeneration [38, 39] and amplified recruitment of neutrophils [11].

It has been shown previously that monomers of rArtinM share their primary structure with the native form of ArtinM (jArtinM), which contributes to its correct folding and exposure of its CRDs, leading to its proper lectin-like activity [25]. This data supports our current findings showing that the ability of rArtinM to activate and degranulate mast cells is dependent on their CRDs.

The fact that rArtinM is expressed as monomers and is able to oligomerize in solution, most likely because of a high monomer concentration, supports our findings, since mast cell activation and degranulation occurred at high concentrations of rArtinM. A similar dose-dependent effect was observed when rArtinM was assayed for its effect on spleen cells. In spleen cells, only high concentrations of rArtinM induced cell proliferation and IL-2 production [40]. Although it shares the sugar-binding specificity of jArtinM, rArtinM differs in its avidity for glycotargets due to its unique quaternary structure. The requirement for high concentrations of rArtinM in order to trigger cellular responses may be associated with its oligomerization upon binding to glycoligands on the cell surface, as has been well established for Galectin 3 [41]. Both IgE [42] and FcεRI [43] are highly glycosylated. IgE contains several mannose residues that could be targets for ArtinM [44, 45]. FcεRI, also presents structural characteristics that could favor its recognition by ArtinM such as several N- glycosylation sites on the FcεRI α subunit [46]. Therefore, rArtinM may be activating mast cells by cross-linking IgE or FcεRI.

However, previous studies have demonstrated that jArtinM is able to degranulate the rat mast cell line, RBL-2H3, as well as peritoneal rat mast cells in an IgE-independent manner [11, 18]. The possibility that rArtinM interacts with other receptors on the mast cell surface, such as TLR4 [6, 47], TLR2 [48, 49], the chemokine receptor CXCR2 [50–52] and complement receptors [7], should not be completely discounted, since degranulation, IL-4 release and the morphological changes on the mast cell surface triggered by rArtinM all occurred in an IgE-independent manner.

It is well established that mast cells can respond in different manners to the same stimulus. For example, FcεRI cross-linking results in NFkB activation leading to mast cell degranulation [53]. However, exposure of IgE sensitized mast cells to low concentrations of specific antigen induces NFAT activation in the absence of degranulation [54]. The same appears to be true for ArtinM. At 10 μg/ml, jArtinM induces mast cell degranulation as well as NFkB and NFAT activation [18], while rArtinM at the same concentration does not induce degranulation. The fact that rArtinM can activate mast cells in a pro-inflammatory manner, without inducing degranulation, makes it an attractive candidate for pharmacological use.

rArtinM was also able to induce IL-4 release. It is known that IL-4 and another cytokines such as, IL-6, VEGF, IL-13 and, TNF-α play a role in allergic inflammatory processes, leading to IgE production by B cells [55, 56]. The ability of rArtinM to induce IL-4 release agrees with our previous data showing that ArtinM can trigger an allergic pro-inflammatory response [11, 18]. However, higher concentrations of rArtinM (20 and 40 μg/ml) were required to trigger responses similar to those observed for jArtinM [18].

## Conclusions

In the current investigation, monomeric rArtinM was able to activate and stimulate mediator release by mast cells through its CRDs. The mechanisms by which rArtinM leads to mast cell activation is dependent on the transcription factor NFkB, but not NFAT. These results demonstrate that the mast cell response depends on the nature and concentration of the stimuli. The present study indicates that rArtinM is a suitable substitute for the native form, since it shares some of the biological activities already described for jArtinM. Therefore, rArtinM may serve as an important and reliable pharmacological agent.

## Methods

### rArtinM preparations

rArtinM was expressed in *Escherichia coli* BL21- CodonPlus(DE3)-RP and purified as previously reported [25]. rArtinM preparations containing less than 0.05 ng/ml of bacterial endotoxin, as determined by the *Limulus amoebocyte* lysate assay, were used in this study (Sigma-Aldrich., St. Louis, MO).

### Size exclusion chromatography

Native and recombinant forms of ArtinM were submitted to size exclusion chromatography for molecular weight determination, on a Superdex 75 column (Sigma Aldrich) coupled to an AKTA protein purification system (GE Healthcare, Uppsala, Sweden), which was calibrated by using protein molecular weight standards (Protein Mixture, GE Healthcare). The molecular weight of proteins was determined by partition coefficient (Kav) using this formula: Kav = Ve-Vo/Vt-Vo, where Ve is the elution volume of the samples, Vt is the total volume and Vo is the void volume of the gel bed. High molecular weight blue dextran was used to determine the void volume.

### Analytical ultracentrifugation

Sedimentation velocity measurements were performed using a Beckman XL-A analytical centrifuge equipped with both absorbance and interference optics. All data were acquired at a rotor-speed of 50,000 rpm at 20 °C using a Beckman An60Ti rotor. For each sample, 100 scans were acquired at 120 s intervals. Buffer density and viscosity as well as the partial specific volume of the protein were calculated using SEDNTERP (Alliance Protein Laboratories, Thousand Oaks, CA,).

### Glycan array analysis

The native and recombinant ArtinM forms were biotinylated as previously described [38] and quantified by determining their absorbance at 280 nm (OD280). Microarrays were composed of lipid-linked oligosaccharide probes robotically printed in duplicate on nitrocellulose-coated glass slides at 2 and 7 fmol per spot (in-house designation sets 18–21bis) using a non-contact instrument, as previously described [57]. The microarray binding assays of biotinylated ArtinM proteins were performed at 19 °C–20 °C, as previously described [58]. In brief, the slide arrays were blocked with 1 % w/v bovine serum albumin (Sigma Aldrich) in casein blocking solution (Pierce Chemical Co, Thermo Fisher, Waltham, MA) for 1 h. The biotinylated ArtinM (50 μg/mL) was overlaid, and binding was detected with streptavidin conjugated to Alexa Fluor 647 (Molecular Probes, Thermo Fisher, Waltham, MA) at 1 μg/mL in blocking solution. Glycoarray data analysis was performed with dedicated software [59]. The binding signals were probe-dose dependent.

### Cells

RBL-2H3 cells, a rat mast cell line, were used in this study [60]. The stable transgenic RBL-2H3 derived cell lines, VB9 and NFkB 2, were also used. The VB9 cell line is a GFP- reporter cell line for NFAT activation [54]. The NFkB 2 cell line is a GFP-reporter cell line for NFkB activation [61], which presents a genome transduction with a reporter vector that possess 4 copies of the binding site for NFkB that regulate GFP expression. All cells were grown as monolayers in Dulbecco’s modified Eagle’s medium (DMEM) (Gibco, Thermo Fisher, Waltham, MA) supplemented with 15 % fetal calf serum (Sigma-Aldrich) as previously described [60]. Transfected cells were selected with geneticin (0.4 mg/ml) (Sigma-Aldrich). All cell lines were generously provided by Dr. Reuben P. Siraganian, NIDCR, NIH, Bethesda, MD.

### Cell sensitization and stimulation

As a positive control for FcεRI stimulated cells, the cells were cultured in the presence of a 1:5000 dilution of mouse IgE anti-TNP ascites fluid (kindly provided by Dr. Reuben P. Siraganian) for 16 h, and then stimulated with the multivalent specific antigen DNP_48_-HSA (Sigma-Aldrich) at 50 ng/ml. As positive control for FcεRI-independent stimulation, the cells were incubated with calcium ionophore-A23187 (Sigma-Aldrich) at 0.1 μM for degranulation assays and, at 0.7 μM for NFkB and NFAT activation assays. In experimental conditions, the cells were sensitized or not with IgE anti-TNP ascites fluid (1:5000) for 16 h and then stimulated with rArtinM. In some experiments, rArtinM was preincubated with D-mannose 100 mM (Sigma-Aldrich) for 1 h at 4 °C. To evaluate release of preformed mediators (β-hexosaminidase and TNF-α) and the lipid mediator LTC4, cells were stimulated for 45 min. For scanning electron microscopy, the cells were stimulated for 20 min. For newly-synthesized IL-4, the cells were stimulated for 12 h. For NFkB and NFAT activation, the cells were stimulated for 5 and 17 h respectively.

### β-Hexosaminidase activity

3.0 × 10^4^ cells/well were plated in 96 well tissue culture plates (Corning Life Sciences, Lowell, MA) in the absence or presence of IgE and cultured overnight. The cells were washed 2 times with Tyrode’s buffer (137 mM NaCl; 2.7 mM KCl; 12 mM NaHCO_3_; 0.37 mM NaH_2_PO_4_; 0.1 mM MgCl_2_; 1.3 mM CaCl_2_; 10 mM Hepes, pH 7.3) supplemented with 0.1 % BSA (Sigma-Aldrich) and 0.01 % gelatin (Sigma-Aldrich) and then incubated with the stimulus diluted in Tyrode’s buffer for 45 min as described above in **Cell sensitization and stimulation**. After stimulation, the supernatants were transferred to clean wells and β-hexosaminidase activity measured as previously described [1]. All assays were run in triplicate.

### Leukotriene C4 and cytokine detection assays

IgE-sensitized or unsensitized cells were incubated with rArtinM for 45 min or 12 h. The concentrations of LTC4, TNF-α and IL-4 in the cell culture supernatants were measured by ELISA (Leukotriene C4 EIA kit, Cayman Chemical Company, MI, USA; OPTEIA™ Rat TNF ELISA kit II; OPTEIA™ Rat IL-4 ELISA kit II, BD Biosciences, San Jose, CA) according to the manufacturer’s instructions.

### Scanning electron microscopy (SEM)

Cells (3x10^4^) were cultured on 13 mm diameter glass coverslips in 24 well plates (Corning Life Sciences). The cells were cultured in the presence or absence of IgE for 16 h. The cells were then stimulated as described in **Cell sensitization and stimulation**. The samples were prepared as previously described [62], and were examined with a JEOL JSM-6610 LV scanning electron microscope (JEOL, Ltd.; Tokyo, Japan).

### Flow cytometric measurements of NFkB and NFAT activation

NFkB2 and VB9 cells (1x10^5^) were sensitized or not with IgE, and then stimulated. Fluorescence levels were measured using a Guava Personal Cell Analysis-96 System and data were processed by Guava InCyte Software (Millipore Co., Billerica, MA).

### Statistics

Data was analyzed using GraphPad Prism (GraphPad Software, Inc., La Jolla, CA). Results were expressed as mean ± SEM. Differences between groups were assessed by one-way ANOVA followed by Tukey’s Multiple Comparison Test. *p < 0.05; **p < 0.01; ***p < 0.001.

## Abbreviations

CRD, carbohydrate recognition domain; TNF-α, tumor necrosis factor alpha; IgE, immunoglobulin E; NFkB, nuclear factor kappa B; NFAT, nuclear factor of activated T-cells; RBL-2H3, rat basophilic leukemia cell line; CXCR2, chemokine receptor 2; HRP, horseradish peroxidase; TLR2, toll like receptor 2; AUC, analytical ultracentrifugation; LTC4, leukotriene C 4; SEM, scanning electron microscopy; FcεRI, high affinity receptor for IgE; VEGF, vascular endothelial growth factor; kD, kilodaltons; MW, molecular weight

## References

[1] Jamur MC, Grodzki AC, Berenstein EH, Hamawy MM, Siraganian RP, Oliver C (2005). Identification and characterization of undifferentiated mast cells in mouse bone marrow. Blood.

[2] Kitamura Y, Go S, Hatanaka K (1978). Decrease of mast cells in W/Wv mice and their increase by bone marrow transplantation. Blood.

[3] Kitamura Y, Shimada M, Go S, Matsuda H, Hatanaka K, Seki M (1979). Distribution of mast-cell precursors in hematopoeitic and lymphopoietic tissues of mice. J Exp Med.

[4] Yuen E, Brown RD, van der Lubbe L, Rickard KA, Kronenberg H (1988). Identification and characterization of human hemopoietic mast cell colonies. Exp Hematol.

[5] Jamur MC, Oliver C (2011). Origin, maturation and recruitment of mast cell precursors. Front Biosci.

[6] Rao KN, Brown MA (2008). Mast cells: multifaceted immune cells with diverse roles in health and disease. Ann N Y Acad Sci.

[7] Galli SJ, Nakae S, Tsai M (2005). Mast cells in the development of adaptive immune responses. Nat Immunol.

[8] Stone KD, Prussin C, Metcalfe DD (2010). IgE, mast cells, basophils, and eosinophils. J Allergy Clin Immunol.

[9] da Silva EZ, Jamur MC, Oliver C (2014). Mast cell function: a new vision of an old cell. J Histochemistry Cytochemistry.

[10] Weller CL, Collington SJ, Williams T, Lamb JR (2011). Mast cells in health and disease. Clin Sci (Lond).

[11] Moreno AN, Jamur MC, Oliver C, Roque-Barreira MC (2003). Mast cell degranulation induced by lectins: effect on neutrophil recruitment. Int Arch Allergy Immunol.

[12] Nakae S, Suto H, Iikura M, Kakurai M, Sedgwick JD, Tsai M, Galli SJ (2006). Mast cells enhance T cell activation: importance of mast cell costimulatory molecules and secreted TNF. J Immunol.

[13] Metz M, Grimbaldeston MA, Nakae S, Piliponsky AM, Tsai M, Galli SJ (2007). Mast cells in the promotion and limitation of chronic inflammation. Immunol Rev.

[14] Pramod SN, Venkatesh YP, Mahesh PA (2007). Potato lectin activates basophils and mast cells of atopic subjects by its interaction with core chitobiose of cell-bound non-specific immunoglobulin E. Clin Exp Immunol.

[15] Wyczolkowska J, Rydzynski K, Prouvost-Danon A (1992). Concanavalin A-induced activation of hamster mast cells: morphological changes and histamine secretion. Int Arch Allergy Immunol.

[16] Frigeri LG, Zuberi RI, Liu FT (1993). Epsilon BP, a beta-galactoside-binding animal lectin, recognizes IgE receptor (Fc epsilon RI) and activates mast cells. Biochemistry.

[17] de Almeida Buranello PA, Moulin MR, Souza DA, Jamur MC, Roque-Barreira MC, Oliver C (2010). The lectin ArtinM induces recruitment of rat mast cells from the bone marrow to the peritoneal cavity. PLoS One.

[18] Barbosa-Lorenzi VC, Buranello PA, Roque-Barreira MC, Jamur MC, Oliver C, Pereira-da-Silva G (2011). The lectin ArtinM binds to mast cells inducing cell activation and mediator release. Biochem Biophys Res Commun.

[19] Ganiko L, Martins AR, Freymuller E, Mortara RA, Roque-Barreira MC (2005). Lectin KM + −induced neutrophil haptotaxis involves binding to laminin. Biochim Biophys Acta.

[20] Pereira-da-Silva G, Moreno AN, Marques F, Oliver C, Jamur MC, Panunto-Castelo A, Roque-Barreira MC (2006). Neutrophil activation induced by the lectin KM+ involves binding to CXCR2. Biochim Biophys Acta.

[21] Toledo KA, Scwartz C, Oliveira AF, Conrado MC, Bernardes ES, Fernandes LC, Roque-Barreira MC, Pereira-da-Silva G, Moreno AN (2009). Neutrophil activation induced by ArtinM: release of inflammatory mediators and enhancement of effector functions. Immunol Lett.

[22] Panunto-Castelo A, Souza MA, Roque-Barreira MC, Silva JS (2001). KM(+), a lectin from Artocarpus integrifolia, induces IL-12 p40 production by macrophages and switches from type 2 to type 1 cell-mediated immunity against Leishmania major antigens, resulting in BALB/c mice resistance to infection. Glycobiology.

[23] Coltri KC, Oliveira LL, Pinzan CF, Vendruscolo PE, Martinez R, Goldman MH, Panunto-Castelo A, Roque-Barreira MC (2008). Therapeutic administration of KM+ lectin protects mice against Paracoccidioides brasiliensis infection via interleukin-12 production in a toll-like receptor 2-dependent mechanism. Am J Pathol.

[24] daSilva LL, de Molfetta-Machado JB, Panunto-Castelo A, Denecke J, Goldman GH, Roque-Barreira MC, Goldman MH. cDNA cloning and functional expression of KM+, the mannose-binding lectin from Artocarpus integrifolia seeds. Biochim Biophys Acta. 2005;1726(3):251–60. 10.1016/j.bbagen.2005.09.006 16242845

[25] Pranchevicius MC, Oliveira LL, Rosa JC, Avanci NC, Quiapim AC, Roque-Barreira MC, Goldman MH (2012). Characterization and optimization of ArtinM lectin expression in Escherichia coli. BMC Biotechnol.

[26] Pesquero NC, Pedroso MM, Watanabe AM, Goldman MH, Faria RC, Roque-Barreira MC, Bueno PR (2010). Real-time monitoring and kinetic parameter estimation of the affinity interaction of jArtinM and rArtinM with peroxidase glycoprotein by the electrogravimetric technique. Biosens Bioelectron.

[27] Coltri KC, Oliveira LL, Ruas LP, Vendruscolo PE, Goldman MH, Panunto-Castelo A, Roque-Barreira MC (2010). Protection against Paracoccidioides brasiliensis infection conferred by the prophylactic administration of native and recombinant ArtinM. Med Mycol.

[28] Rosa JC, De Oliveira PS, Garratt R, Beltramini L, Resing K, Roque-Barreira MC, Greene LJ (1999). KM+, a mannose-binding lectin from Artocarpus integrifolia: amino acid sequence, predicted tertiary structure, carbohydrate recognition, and analysis of the beta-prism fold. Protein Science.

[29] Cecilio NT, Carvalho FC, Liu Y, Moncrieffe M, Buranello PA, Zorzetto-Fernandes AL, Luche DD, Hanna ES, Soares SG, Feizi T (2016). Yeast expressed ArtinM shares structure, carbohydrate recognition, and biological effects with native ArtinM. Int J Biol Macromol.

[30] Pratap JV, Jeyaprakash AA, Rani PG, Sekar K, Surolia A, Vijayan M (2002). Crystal structures of artocarpin, a Moraceae lectin with mannose specificity, and its complex with methyl-alpha-D-mannose: implications to the generation of carbohydrate specificity. J Mol Biol.

[31] Misquith S, Rani PG, Surolia A (1994). Carbohydrate binding specificity of the B-cell maturation mitogen from Artocarpus integrifolia seeds. J Biol Chem.

[32] Bachelet I, Levi-Schaffer F (2007). Mast cells as effector cells: a co-stimulating question. Trends Immunol.

[33] Gordon JR, Galli SJ (1991). Release of both preformed and newly synthesized tumor necrosis factor alpha (TNF-alpha)/cachectin by mouse mast cells stimulated via the Fc epsilon RI. A mechanism for the sustained action of mast cell-derived TNF-alpha during IgE-dependent biological responses. J Exp Med.

[34] McLachlan JB, Hart JP, Pizzo SV, Shelburne CP, Staats HF, Gunn MD, Abraham SN (2003). Mast cell-derived tumor necrosis factor induces hypertrophy of draining lymph nodes during infection. Nat Immunol.

[35] Gordon JR, Galli SJ (1990). Mast cells as a source of both preformed and immunologically inducible TNF-alpha/cachectin. Nature.

[36] Lauterbach M, O’Donnell P, Asano K, Mayadas TN (2008). Role of TNF priming and adhesion molecules in neutrophil recruitment to intravascular immune complexes. J Leukoc Biol.

[37] Ott VL, Cambier JC, Kappler J, Marrack P, Swanson BJ (2003). Mast cell-dependent migration of effector CD8+ T cells through production of leukotriene B4. Nat Immunol.

[38] Pinto-da-Silva LLP-C A, De-Souza-Goldman MH, Roque-Antunes-Barreira MC, De-Oliveira RS, Dias-Baruffi M, Blanco-de-Molfeta-Machado J. In: WIPO, editor. Pharmaceutical Composition Comprising Lectin, vol. WO2004100861. Brasil: 2004.

[39] Chahud F, Ramalho LN, Ramalho FS, Haddad A, Roque-Barreira MC (2009). The lectin KM+ induces corneal epithelial wound healing in rabbits. Int J Exp Pathol.

[40] Silva TA, Souza MA, Cecilio NT, Roque-Barreira MC (2014). Activation of spleen cells by ArtinM may account for its immunomodulatory properties. Cell Tissue Res.

[41] Demetriou M, Granovsky M, Quaggin S, Dennis JW (2001). Negative regulation of T-cell activation and autoimmunity by Mgat5 N-glycosylation. Nature.

[42] Shade KT, Platzer B, Washburn N, Mani V, Bartsch YC, Conroy M, Pagan JD, Bosques C, Mempel TR, Fiebiger E (2015). A single glycan on IgE is indispensable for initiation of anaphylaxis. J Exp Med.

[43] LaCroix EL, Froese A (1993). The N-linked oligosaccharides of the Fc epsilon receptors of rat basophilic leukemia cells. Mol Immunol.

[44] Dorrington KJ, Bennich HH (1978). Structure-function relationships in human immunoglobulin E. Immunol Rev.

[45] Arnold JN, Radcliffe CM, Wormald MR, Royle L, Harvey DJ, Crispin M, Dwek RA, Sim RB, Rudd PM (2004). The glycosylation of human serum IgD and IgE and the accessibility of identified oligomannose structures for interaction with mannan-binding lectin. J Immunol.

[46] Ravetch JV, Kinet JP (1991). Fc receptors. Annu Rev Immunol.

[47] Gon Y, Nunomura S, Ra C (2005). Common and distinct signalling cascades in the production of tumour necrosis factor-alpha and interleukin-13 induced by lipopolysaccharide in RBL-2H3 cells. Clin Exp Allergy.

[48] Carlos D, Frantz FG, Souza-Junior DA, Jamur MC, Oliver C, Ramos SG, Quesniaux VF, Ryffel B, Silva CL, Bozza MT (2009). TLR2-dependent mast cell activation contributes to the control of Mycobacterium tuberculosis infection. Microbes Infection/Institut Pasteur.

[49] Saluja R, Delin I, Nilsson GP, Adner M (2012). FcepsilonR1-mediated mast cell reactivity is amplified through prolonged Toll-like receptor-ligand treatment. PLoS One.

[50] Nasser MW, Raghuwanshi SK, Grant DJ, Jala VR, Rajarathnam K, Richardson RM (2009). Differential activation and regulation of CXCR1 and CXCR2 by CXCL8 monomer and dimer. J Immunol.

[51] Raghuwanshi SK, Su Y, Singh V, Haynes K, Richmond A, Richardson RM (2012). The chemokine receptors CXCR1 and CXCR2 couple to distinct G protein-coupled receptor kinases to mediate and regulate leukocyte functions. J Immunol.

[52] Juremalm M, Nilsson G (2005). Chemokine receptor expression by mast cells. Chem Immunol Allergy.

[53] Klemm S, Ruland J (2006). Inflammatory signal transduction from the Fc epsilon RI to NF-kappa B. Immunobiology.

[54] Grodzki AC, Moon KD, Berenstein EH, Siraganian RP (2009). FcepsilonRI-induced activation by low antigen concentrations results in nuclear signals in the absence of degranulation. Mol Immunol.

[55] He SH, Zhang HY, Zeng XN, Chen D, Yang PC (2013). Mast cells and basophils are essential for allergies: mechanisms of allergic inflammation and a proposed procedure for diagnosis. Acta Pharmacol Sin.

[56] Hu ZQ, Zhao WH, Shimamura T (2007). Regulation of mast cell development by inflammatory factors. Curr Med Chem.

[57] Palma AS, Feizi T, Zhang Y, Stoll MS, Lawson AM, Diaz-Rodriguez E, Campanero-Rhodes MA, Costa J, Gordon S, Brown GD (2006). Ligands for the beta-glucan receptor, Dectin-1, assigned using “designer” microarrays of oligosaccharide probes (neoglycolipids) generated from glucan polysaccharides. J Biol Chem.

[58] Liu Y, Childs RA, Palma AS, Campanero-Rhodes MA, Stoll MS, Chai W, Feizi T (2012). Neoglycolipid-based oligosaccharide microarray system: preparation of NGLs and their noncovalent immobilization on nitrocellulose-coated glass slides for microarray analyses. Methods Mol Biol.

[59] Stoll MS (2009). Software tools for sorting, processing and displaying carbohydrate microarray data. Beilstein Symposium on Glyco-Bioinformatics, 4–8 October, 2009: 2009; Potsdam, Germany.

[60] Barsumian EL, Isersky C, Petrino MG, Siraganian RP (1981). IgE-induced histamine release from rat basophilic leukemia cell lines: isolation of releasing and nonreleasing clones. Eur J Immunol.

[61] Valim CX, da Silva EZ, Assis MA, Fernandes FF, Coelho PS, Oliver C, Jamur MC (2015). rPbPga1 from Paracoccidioides brasiliensis Activates Mast Cells and Macrophages via NFkB. PLoS Negl Trop Dis.

[62] Marchini-Alves CM, Nicoletti LM, Mazucato VM, de Souza LB, Hitomi T, Alves Cde P, Jamur MC, Oliver C (2012). Phospholipase D2: a pivotal player modulating RBL-2H3 mast cell structure. J Histochemistry Cytochemistry.

